# LRCH1 suppresses migration of CD4^+^ T cells and refers to disease activity in ulcerative colitis

**DOI:** 10.7150/ijms.39106

**Published:** 2020-02-17

**Authors:** Yibo Wang, Hairong Zhang, Heng He, Kuankuan Ai, Wei Yu, Xiao Xiao, Yufen Qin, Lingming Zhang, Huabao Xiong, Guangxi Zhou

**Affiliations:** 1 Department of Gastroenterology, Affiliated Hospital of Jining Medical University, Jining Medical University, Jining, Shandong 272000, P.R. China.; 2 Institute of Immunology and Molecular Medicine, Jining Medical University, Jining, Shandong 272000, P.R. China.

**Keywords:** LRCH1, ulcerative colitis, CD4^+^ T cells, migration

## Abstract

**Background:** Ulcerative colitis (UC) is a chronically remittent and progressive inflammatory disorder. LRCH1 is reported to be involved in the immune-regulation of several diseases. However, the exact roles of LRCH1 in UC are still obscure.

**Materials and Methods:** LRCH1 expression was analyzed in the inflamed mucosa and peripheral blood mononuclear cells (PBMCs) from patients with UC by quantitative RT-PCR and immunohistochemistry. Peripheral blood CD4^+^ T cells were transfected with lentivirus-expressing LRCH1 (LV-LRCH1) or LV-sh-LRCH1, and cytokine expression was determined by using flow cytometry, quantitative RT-PCR and ELISA. Transfected CD4^+^ T cells were harvested to examine the capacity of chemotaxis using Transwell plate.

**Results:** LRCH1 expression was highly decreased in colonic mucosa and PBMCs from patients with A-UC, and negatively correlated with disease activity. Up or down regulation of LRCH1 did not affect the differentiation of CD4^+^ T cells, and the related cytokines expression. Moreover, LRCH1 inhibited migratory capacity of CD4^+^ T cells toward CXCL12 by PKCα.

**Conclusion:** LRCH1 plays an important role in the pathogenesis of UC, possibly through modulating the migration of CD4^+^ T cells. Therefore, targeting LRCH1 might serve as a novel therapeutic approach in the management of UC.

## Introduction

Ulcerative colitis (UC), one subtype belonging to inflammatory bowel diseases (IBD), is one chronically relapsing and remitting inflammatory disorder of the gastrointestinal tract that have an increasing incidence, resulting in high socio-economic burden 1-3. Currently, the etiology and pathogenesis of UC are considered as the abnormal immune responses to microbial antigen in genetically susceptible individuals 4, 5. During intestinal mucosa inflammation, large amounts of innate or adaptive immune cells (e.g., neutrophils, macrophages, CD4^+^ T cells, B cells, monocytes) are recruited into intestinal lamina propria, and play a role in pathological immune responses 6-8.

It is well known that CD4^+^ T cells mediated inflammatory immune responses have been implicated in the pathogenesis of UC. UC is characterized by massive infiltration of effector T cells into the mucosa associated with T cell dysfunction 9, 10. Previous studies have found that frequencies of CD62L^+^ T lymphocytes were increased in the lamina propria in UC patients, and rapid migration of thymic emigrants to the colonic mucosa was found in UC patients 11. Increased proliferation of CD4^+^ T cells aggravated intestinal colitis in Rag^-/-^ mice 12. Under different activation and stimulation of signaling pathways, naïve CD4^+^ T cells differentiated into helper T (Th) cells, termed Th1, Th2 and Th17, and induced regulatory T cells (Treg), which suppressed the effector functions of other types of Th cell 13. Our previous studies have demonstrated that abnormal Th1 and Th17 immune responses promoted colonic mucosal inflammation, whereas Treg cells possessed the suppressive activity, characterized by high levels of IL-10 and TGF-β 14, 15. It is reported that Th1 cells were highly enriched in the inflamed mucosa of IBD patients, and downregulation of CXCR3 in Th1 cells interfered with the migration of Th1 cells into the colonic mucosa and protected mice against severe acute and relapsing intestinal inflammation 16.

Leucine-rich repeat (LRRs) and calponin homology (CH) domain-containing proteins (LRCH) are characterized by a unique combination of protein domains that are otherwise common in eukaryotes, the LRR and CH domain 17. LRCH family is comprised of just one protein (dLRCH) in Drosophila melanogaster and four (LRCH1-4) in both mouse and human. LRCH proteins function as cytoskeletal regulators during cell division 17. LRCH1 is one of the members of LRCH family, and is reported to be a risk factor for knee osteoarthritis 18. A stratified GWAS meta-analysis demonstrated that a variant in the LRCH1 gene (rs754106) was associated with hip osteoarthritis 19. Studies also found that LRCH1 functioned as a negative regulator of Cdc42 activation, and participated in the pathogenesis in experimental autoimmune encephalomyelitis 20. These data suggest that LRCH1 are involved in the immune-regulation of several diseases, however, the roles of LRCH1 in UC are still obscure.

In this study, we found that LRCH1 expression was highly decreased in colonic mucosa and peripheral blood mononuclear cells (PBMCs) from patients with UC, and negatively correlated with disease activity. Up or down regulation of LRCH1 did not affect the differentiation of CD4^+^ T cells, and the related cytokines expression. Moreover, we found that LRCH1 inhibited migration of CD4^+^ T cells toward CXCL12 by PKCα. Collectively, our data suggest that LRCH1 plays an important role in the pathogenesis of UC, possibly through modulating the migration of CD4^+^ T cells. Therefore, LRCH1 might serve as a therapeutic target for treatment of human UC.

## Materials and Methods

### Patients

All UC patients included in this study were recruited at the Department of Gastroenterology, Affiliated Hospital of Jining Medical college (Jining, Shandong, China) from July 2017 to May 2019. The diagnosis of UC was established by the combination of clinical symptoms, radiological findings, endoscopic examination and histological features 21. The disease severity of UC patients was evaluated according to international standard criteria such as Mayo index. Slight UC was defined by 3-5 Mayo index, mild UC was 6-10 Mayo index, and severe UC was 11-12 Mayo index 22. Intestinal mucosal lesions of UC patients were graded by the ulcerative colitis endoscopic index of severity (UCEIS) by colonoscopy 23. Colonic biopsies were collected from 30 patients with UC who underwent endoscopy, and 23 healthy controls (HC) who underwent endoscopy for routine physical examinations. EDTA anticoagulated blood samples were taken from 33 patients with UC and 26 healthy volunteers. The baseline clinical characteristics of these patients were listed in Supplementary Table 1. This study was approved by the Institutional Review Board for Clinical Research of Affiliated Hospital of Jining Medical college. Written informed consent was obtained from each participant before initiating the study protocol.

### Reagents

Anti-human LRCH1 mAb was purchased from Novus (Littleton, CO, USA). Anti-human CD3, anti-human CD28 was purchased from BD (San Diego, CA, USA). Anti-human CD4 magnetic particles were purchased from BD (San Diego, CA, USA). Enzyme-linked immunosorbent assay (ELISA) kits for human TNF-α, IL-17A, IFN-γ, IL-4, and IL-10 were purchased from BioLegend (San Diego, CA, USA). Fluorochrome-conjugated anti-human CD4, IFN-γ, IL-17A, IL-4 and Foxp3 were purchased from BD (San Diego, CA, USA). Recombinant CXCL12 was purchased from R&D Systems (Minneapolis, MN, USA). Phorbol-12-myristate-13-acetate (PMA) and ionomycin were purchased from Sigma-Aldrich (St. Louis, MO, USA). Penicillin, streptomycin, 4-(2-hydroxyethyl) piperazine-1-ethanesulfonic acid (HERPS), sodium pyruvate and 2-mercaptoethanol (2-ME) were purchased from Life Technologies (Carlsbad, CA, USA).

### Isolation of peripheral blood mononuclear cells (PBMCs)

As described previously 24, EDTA anti-coagulated blood (about 10 mL) was diluted with PBS (1:1). After density centrifugation using FicollPaqueTM Plus (GE Healthcare Bio-Sciences Corp; Piscataway, NJ), PBMCs were collected from the interface and then were washed 3 times in PBS. Viable cells were counted using Trypan Blue exclusion on a hemocytometer.

### Lentivirus-mediated CD4^+^ T-cell transfection

Peripheral blood CD4^+^ T cells were isolated by using anti-human CD4 particles and activated with plate-bound anti-CD3 (5 mg/mL) and anti-CD28 (2 mg/mL) mAbs for 48 h 15. These cells (1×10^5^/well) were transfected with lentivirus-expressing LRCH1 (LV-LRCH1, ID: NM_015116.2) or LV-sh-LRCH1 (target sequence: 5'-GCAGATAGGTCAGTTGAAATC-3') or with either negative control lentiviral vector (LV-NC) according to the manufacturer's protocols (multiplicity of infection = 180, Biolink, Shanghai, China). After centrifugation for 2 h, lentivirus was incubated for 5 h in complete RPMI 1640 medium supplemented with protamine (10 μg/mL) in 24-well plates as our previous study described. After 3 washes with RPMI 1640 medium, transfected cells were resuspended in complete RPMI 1640 medium and then cultured for 5 days. During this period, transfection efficiency was assessed by means of fluorescent microscopy 72 h after transfection 15. These CD4^+^ T cells were harvested for flow cytometry analysis and qRT-PCR. Supernatants also were harvested for ELISA.

### Flow cytometry analysis

Transfected CD4^+^ T cells (1×10^6^/mL) were harvested and then stimulated with PMA (50 ng/mL) and ionomycin (1000 ng/mL) for further 5 h in 24 well plates. Brefeldin A (3 μg/mL) was added into the culture medium for the last 3 h. Intracellular expression of interleukin IL-17A, IFN-γ, IL-4 and Foxp3 was analyzed on a flow cytometry (BD; San Diego, CA, USA).

### Quantitative real-time polymerase chain reaction (qRT-PCR)

Total RNA was extracted from the cells or tissues with a 260/280 ratio between 1.8 to 2.0. The complementary DNA (cDNA) was synthesized with 5×All-in-one RT mastermix (abm) according to the manufacturer's instructions. The synthesized cDNA was stored at -20°C for use. qRT-PCR was performed using SYBR green methodology according to the following conditions: 95°C for 1 min, followed by 40 cycles at 95°C for 15 s and 60°C for 30 s with 40 cycles. All the primers were synthesized from ShengGong BioTeck (Shanghai, China) (Table 1). The relative levels of target gene expression were calculated using the 2^-△△Ct^ method.

### ELISA

The supernatants of CD4^+^ T cell were collected for analysis of concentrations of TNF-α, IFN-γ, IL-17A, IL-4, and IL-10 using ELISA according to the manufacturer's instructions. The diluted supernatants and standard samples were added and incubated for 2 h at 37°C. After thoroughly washed in PBST, the plates were incubated with detection antibodies for 1 h and HRP for 30 mins. Finally, the samples were colored with TMB and the value of OD was detected at 450 nm with a spectrophotometer (BioTek).

### Immunohistochemistry

To localize LRCH1 expression, colonic biopsies from UC patients and healthy donors were fixed and embedded. After incubation with Envision flex peroxidase-blocking reagent for 10 mins, these sections were incubated with rabbit anti-human LRCH1 antibody (dilution 1:200) at 4°C overnight. After washed in PBS, the sections were incubated with HRP-conjugated anti-rabbit at room temperature for 60 mins. The colour reaction was developed with 3, 3′- diaminobenzidine and the sections were counterstained with haematoxylin. As negative controls, sections were treated with PBS instead of primary antibody. The positive cells that stained with anti-LRCH1 antibody were observed under a light microscopy.

### Migration assay of CD4^+^ T cells *in vitro*

Transfected CD4^+^ T cells (1×10^5^) suspended in 100 µL medium were placed into the top well of a Transwell chamber (5 µm, Corning), and 600 μL medium containing human CXCL12 (50 ng/ml) was added into the bottom well. After 4 h of incubation at 37°C, cells in the bottom well were collected and counted.

### Statistical Analysis

Data was expressed as mean ± SEM, and analyzed using SPSS statistics version 20.0 (SPSS, Chicago, IL, USA). Statistical comparisons were performed using an unpaired two-tailed Student's t test or one-way analysis of variance (ANOVA). Pearson's correlation was performed to analyze the correlation of LRCH1 expression and Mayo index, UCEIS, C-reactive protein (CRP), and erythrocyte sedimentation rate (ESR). Statistical significance was set at *p* < 0.05.

## Results

### LRCH1 protein expression is highly decreased in colonic mucosa from patients with active UC

**Figure 1:**
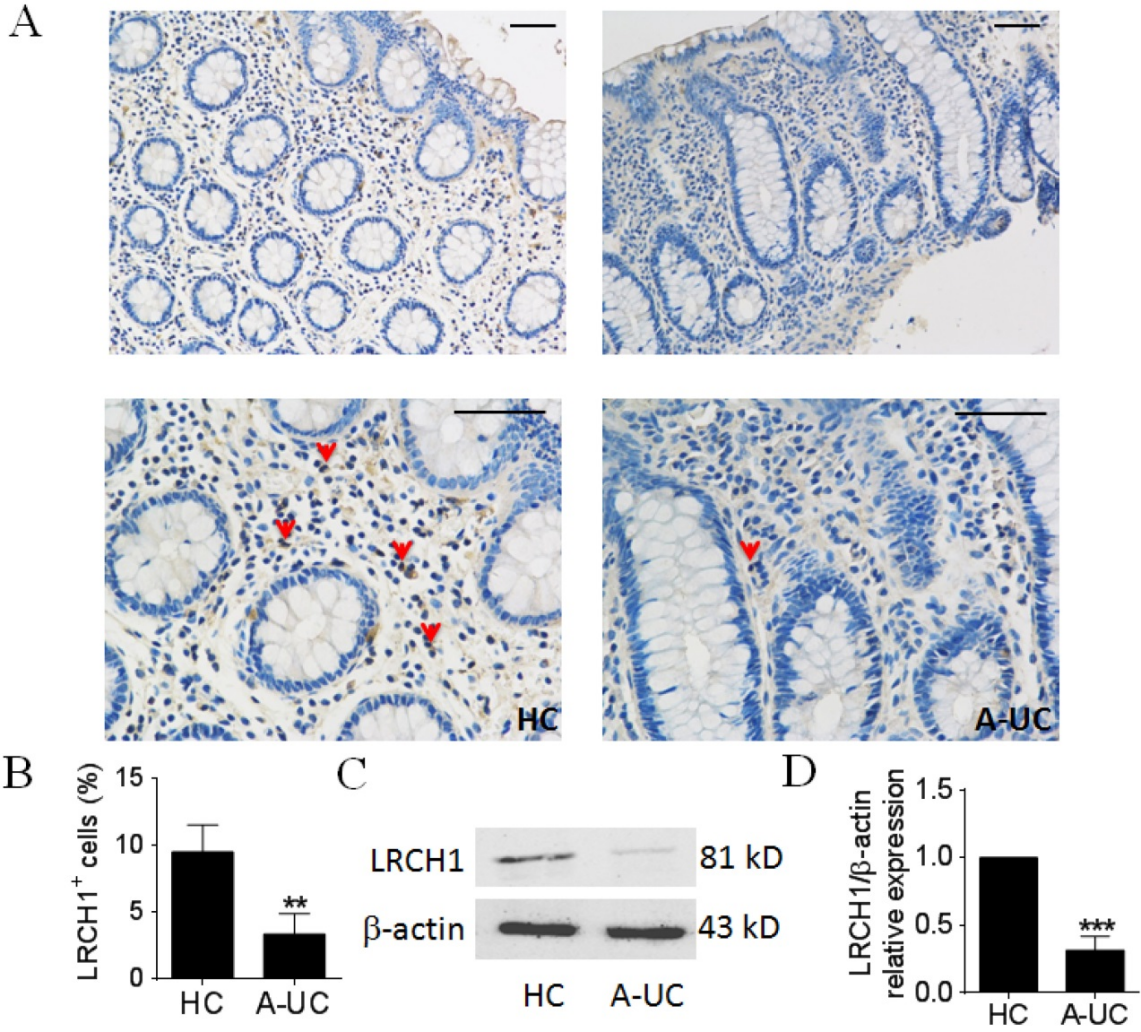
LRCH1 protein expression is highly decreased in colonic mucosa from patients with A-UC. A. Representative images of immunohistochemical staining of LRCH1 in inflamed colon from patients with A-UC (n = 7), and normal colonic mucosa from HC (n = 5). Original magnification ×200 (top) and original magnification ×400 (bottom). Scale bar represents 50 μm. Data are representative of 3 independent experiments. B. Percentages of LRCH1^+^ cells in colonic mucosa in (A) were shown in the bar. **p < 0.01. (C and D) LRCH1 protein expression in colonic mucosa from patients with A-UC (n = 13), and HC (n = 10) was examined by Western blotting, with β-actin as reference. ***p < 0.001.

Previous studies have demonstrated that LRCH1 participated in the pathogenesis of several immune diseases (e.g., osteoarthritis), however, the roles of LRCH1 in UC are still obscure. We collected inflamed colonic mucosa from patients with active UC, and examined LRCH1 protein expression by IHC and Western blot. IHC staining showed that the percentage of LRCH1 positive cells was markedly decreased in lamina propria in inflamed mucosa from patients with UC compared with HC (Figure 1A, B). Then we found that protein expression of LRCH1 in inflamed mucosa from active UC patients was significantly decreased compared with HC (Figure 1C, D). Therefore, our data indicate that LRCH1 may play an important role in the pathogenesis of UC.

### LRCH1 mRNA expression is decreased in inflamed Mucosa and PBMCs in patients with active UC, and correlated with disease activity

**Figure 2:**
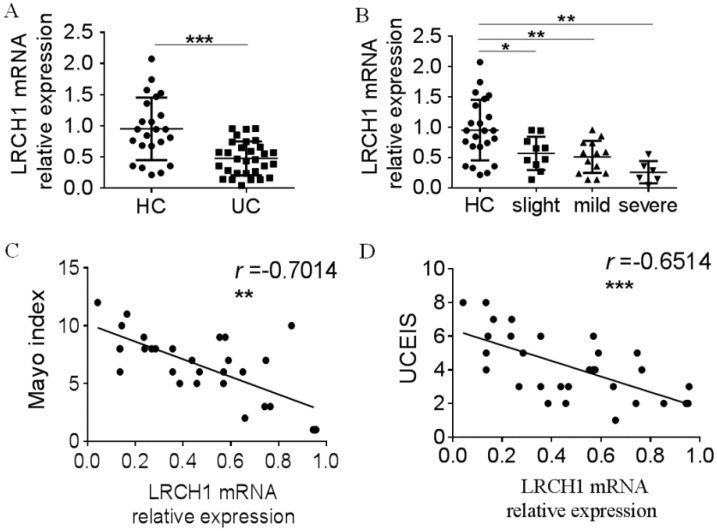
Decreased LRCH1 mRNA expression in colonic mucosa from patients with A-UC is correlated with disease activity. A. LRCH1 mRNA expression in colonic mucosa from patients with UC (n = 30), and HC (n = 23) was examined by qRT-PCR. Gene expression was normalized to GAPDH in each group. ***p < 0.001. B. Disease activity for UC was graded by Mayo index. LRCH1 mRNA expression in colonic mucosa from patients with slight UC (n = 10), mild UC (n = 14), severe UC (n = 6) and HC (n = 23) was analyzed. Gene expression was normalized to GAPDH in each group. *p < 0.05, **p < 0.01. C. Correlation analysis was performed between the Mayo Index and LRCH1 mRNA expression in inflamed mucosa of patients with UC (*r* = -0.7014, **p < 0.01). D. Correlation analysis was performed between UCEIS and LRCH1 mRNA expression in inflamed mucosa of patients with UC (*r* = -0.6514, *** p < 0.001).

To further study the roles of LRCH1 in UC, we extracted the total mRNA from inflamed colonic tissues, and examined LRCH1 mRNA expression by qRT-PCR. We found that expression of LRCH1 mRNA also was significantly lower in affected mucosal from patients with UC than that in HC (Figure 2A). Moreover, we divided these UC patients to slight, mild and severe groups according to Mayo index, and then compared LRCH1 mRNA expression in different groups. We found that there were significant differences of LRCH1 mRNA expression in different groups. The expression in severe group was lowest, and then mild group, slight group (Figure 2B). Therefore, we hypothesized that LRCH1 expression might associated with disease activity in UC patients. Then we performed Pearson's correlation analysis between LRCH1 expression and Mayo index, UCEIS, which were the international standard criteria to evaluate the clinical and endoscopic disease activity in UC patients. We found that LRCH1 expression in inflamed mucosa was significantly correlated with Mayo index and UCEIS in UC (Figure 2C, *r* = -0.7014, *p* < 0.01, Figure 2D, *r* = -0.6514, *p* < 0.001).

**Figure 3:**
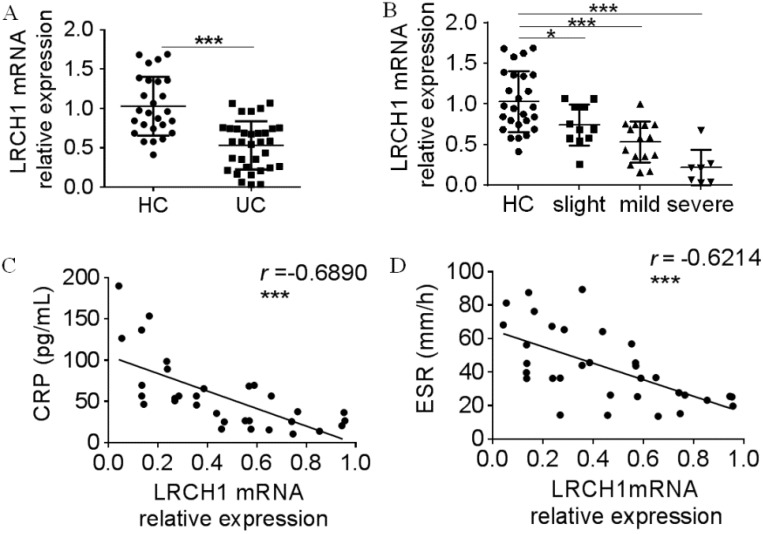
Decreased LRCH1 mRNA expression in PBMCs from patients with A-UC is correlated with disease activity. A. LRCH1 mRNA expression in PBMCs from patients with UC (n = 33), and HC (n = 26) was examined by qRT-PCR. Gene expression was normalized to GAPDH in each group. ***p < 0.001. B. Disease activity for UC was graded by Mayo index. LRCH1 mRNA expression in PBMCs from patients with slight UC (n = 11), mild UC (n = 15), severe UC (n = 7) and HC (n = 26) was analyzed. Gene expression was normalized to GAPDH in each group. *p < 0.05, ***p < 0.001. C. Correlation analysis was performed between CRP and LRCH1 mRNA expression in PBMCs from patients with UC (*r* = -0.6890, ***p < 0.001). D. Correlation analysis was performed between ESR and LRCH1 mRNA expression in PBMCs from patients with UC (*r* = -0.6214, ***p < 0.001).

Moreover, we collected peripheral blood samples from UC patients and HC, and examined LRCH1 mRNA expression in PBMCs. As shown in Figures 3A and 3B, expression of LRCH1 mRNA in PBMCs also was markedly decreased, and the severe group exhibited the lowest expression of LRCH1. CRP and ESR are frequently used to evaluate clinical disease activity in UC 25, and significantly correlation between LRCH1 mRNA expression in PBMCs and CRP, ESR were also observed in patients with UC (Figure 3C, 3 *r* =-0.6890, *p* < 0.001, Figure 3D, *r* =-0.6214, *p* < 0.001). Collectively, our data indicate that LRCH1 expression are associated with disease activity in patients with UC, and might be involved in disease development and progression.

### LRCH1 does not affect the differentiation of CD4^+^ T cells

Immune responses mediated by CD4^+^ T cells are reported to play an important role in the pathogenesis of UC. In this study, we found that LRCH1 was expressed in CD4^+^ T cells, and its expression was significantly decreased in CD4^+^ T cells from UC patients compared with HC (Supplementary Figure 1). Then we investigated whether LRCH1 modulated the differentiation of CD4^+^ T cells. As our study previously described 9, CD4^+^ T cells were isolated from peripheral blood of UC patients and healthy donors, and then transfected with LV-LRCH1 and LV-sh-LRCH1, respectively, which could upregulate or downregulate LRCH1 expression. LV-NC was used as the control. After lentivirus transfection, the survival rate of transfected cells was above 90% examined by flow cytometry (Supplementary Figure 2A), and transfection efficacy was examined by qRT-PCR. As expected, LV-LRCH1 could markedly increase expression of LRCH1 mRNA, and LV-sh-LRCH1 significantly decreased LRCH1 mRNA expression compared with that in controls (Supplementary Figure 2B).

**Figure 4:**
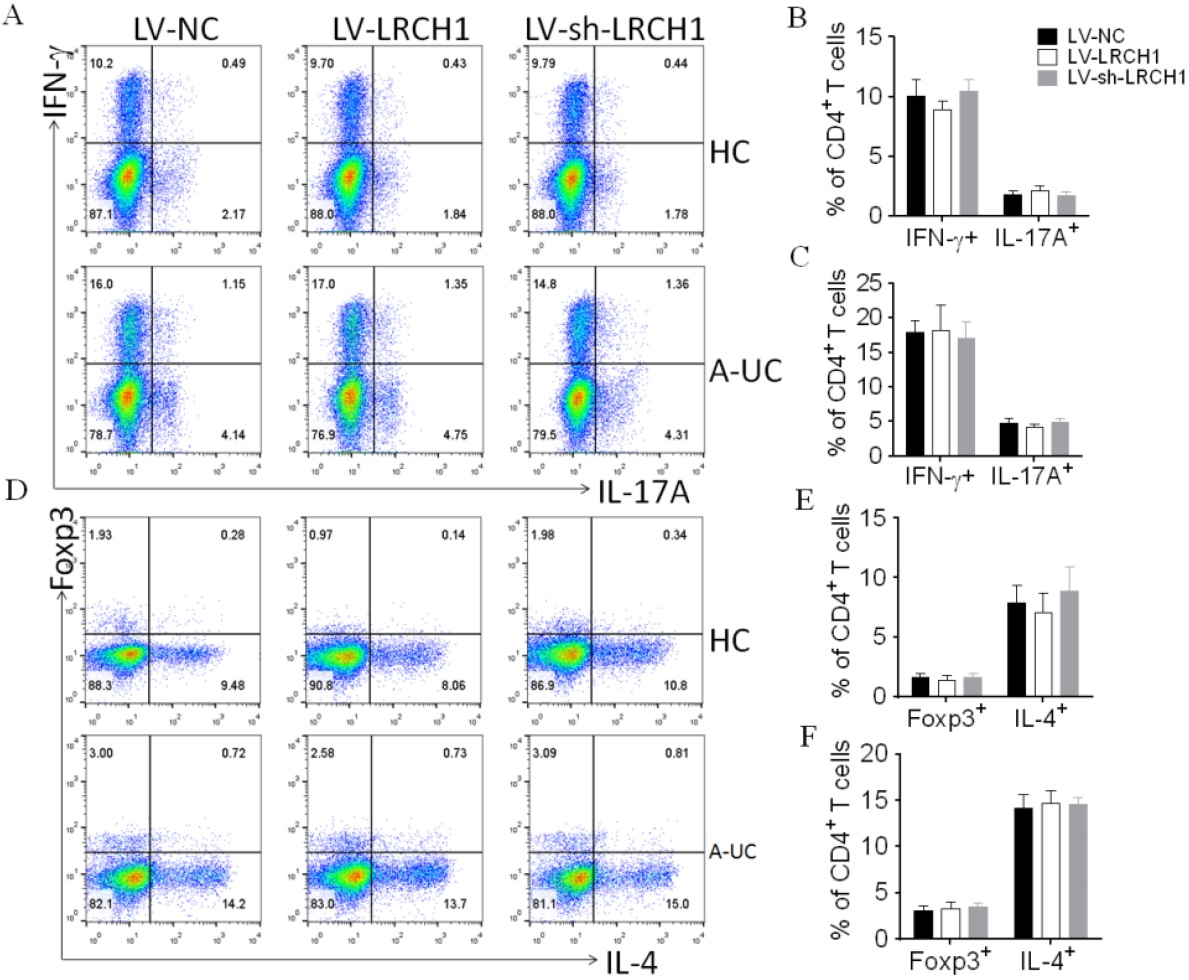
LRCH1 does not affect the differentiation of CD4^+^ T cells. CD4^+^ T cells (1×10^5^/well) were isolated from peripheral blood of patients with A-UC (n = 15), or HC (n = 14), and were first preactivated with anti-CD3 (5 mg/mL) and anti-CD28 (2 mg/mL) mAbs *in vitro* for 48 h and transfected with LV-LRCH1, LV-sh-LRCH1, or LV-NC, respectively, for 5 h. After transfection, CD4^+^ T cells were then cultured under stimulation with anti-CD3 (5 mg/mL) and anti-CD28 (2 mg/mL) mAbs for a further 5 days. On day 5, cultured cells were collected to examine intracellular expression of IL-17A, IFN-γ, IL-4, and Foxp3 by using flow cytometry (A). B - E. Percentages of IFN-γ^+^ and IL-17A^+^CD4^+^ T cells in HC (B), patients with A-UC (C), and Percentages of Foxp3^+^ and IL-4^+^CD4^+^ T cells in HC (D), patients with A-UC (E) were shown in the bar. Data are representative of 3 independent experiments.

Transfected cells were then cultured under the stimulation of anti-CD3 and anti-CD28 mAbs *in vitro*. 5 days later, these cells were collected for the use of flow cytometry or qRT-PCR. As shown in Figure 4A-C, the percentages of IFN-γ^+^ or IL-17A^+^ cells were the same in CD4^+^ T cells transfected with LV-LRCH1 or LV-sh-LRCH1 compared with controls. And upregulation or downregulation of LRCH1 didn't alter the percentages of IL-4^+^ or Foxp3^+^ cells (Figure 4D-F). Then we examined concentrations of TNF-α, IFN-γ, IL-4, IL-17A, and IL-10 in the supernatants by means of ELISA, and found that their levels also were the same in supernatants transfected with LV-LRCH1 or LV-sh-LRCH1 compared with controls (Supplementary Figure 3A-E). Furthermore, mRNA levels of T-bet, GATA3, RORC, and Foxp3 were examined by means of qRT-PCR. As demonstrated in Supplementary Figure 3F-I, their levels also didn't alter in CD4^+^ T cells after transfected with LV-LRCH1 or LV-sh-LRCH1. Taken together, these data suggest that LRCH1 does not participate in the modulation of CD4^+^ T cells differentiation in the pathogenesis of UC.

### LRCH1 inhibits migration of CD4^+^ T cells

**Figure 5:**
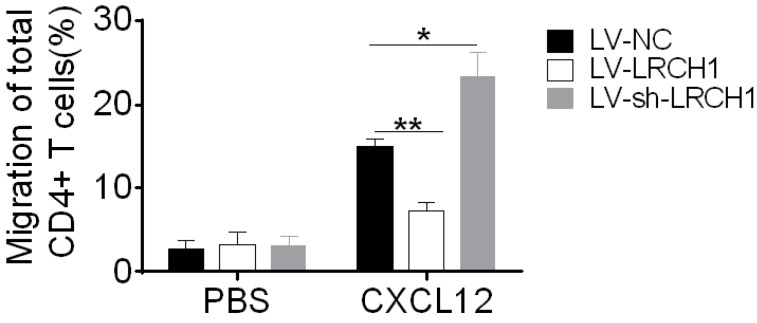
LRCH1 inhibits the migration of CD4^+^ T cells. CD4^+^ T cells (1×10^5^/well) were isolated from peripheral blood of patients with A-UC (n = 10), and transfected with LV-LRCH1, LV-sh-LRCH1, or LV-NC, respectively as shown in Figure 4. On day 3, transfected cells were collected and used to the chemotaxis assay. CD4^+^ T cells (1×10^5^) were suspended in 100 µL medium were placed into the top well of a transwell chamber (5 µm, Corning), and 600 μL medium containing human CXCL12 (50 ng/ml) was added into the bottom well. After 4 h of incubation, cells in the bottom well were collected and counted. *p < 0.05, **p < 0.01. Data are representative of 3 independent experiments.

Colonic mucosal inflammation in UC is characterized by massive infiltration of effector T cells into the mucosa associated with T-cell dysfunction. In this study, we found that decreased or increased LRCH1 expression didn't affect differentiation of CD4^+^ T cells, so we then examined whether LRCH1 modulated the proliferation and apoptosis of CD4^+^ T cells. However, LRCH1 was not involved in the process of proliferation and apoptosis in CD4^+^ T cells (Supplementary Figure 4). Then we found that migratory capacity of CD4^+^ T cells from UC patients was higher than that from HC (Supplementary Figure 5A). We hypothesized that LRCH1 might participated in the regulation of CD4^+^ T cells migration. To this end, peripheral CD4^+^ T cells were isolated and transfected with LV-LRCH1 or LV-sh-LRCH1, and then migratory capacity of CD4^+^ T cells was analyzed in Transwell plate. As shown in Figure 5, upregulation of LRCH1 significantly suppressed the migration of CD4^+^ T cells toward CXCL12 compared with controls. And oppositely, the migratory capacity was markedly enhanced in CD4^+^ T cells transfected of LV-sh-LRCH1, which could downregulate LRCH1 expression. Therefore, our data indicate that LRCH1 suppresses the migratory capacity of CD4^+^ T cells in patients with UC.

### LRCH1 inhibits CD4^+^ T cells migration by PKCα

We then investigated how LRCH1 regulated migration of CD4^+^ T cells. To this end, we examined expression of CXCR4 in CD4^+^ T cells transfected with LV-LRCH1 or LV-sh-LRCH1, which was an chemokine receptor expressed in T cells, and involved in the modulation of CD4^+^ T cells migration 26. However, there was no significantly difference of CXCR4 expression between CD4^+^ T cells transfected with LV-LRCH1 or LV-sh-LRCH1 and controls (Supplementary Figure 5B), which suggested that LRCH1 did not regulate CXCR4 expression, and might be through other mechanisms in the regulation of CD4^+^ T cells migration.

**Figure 6:**
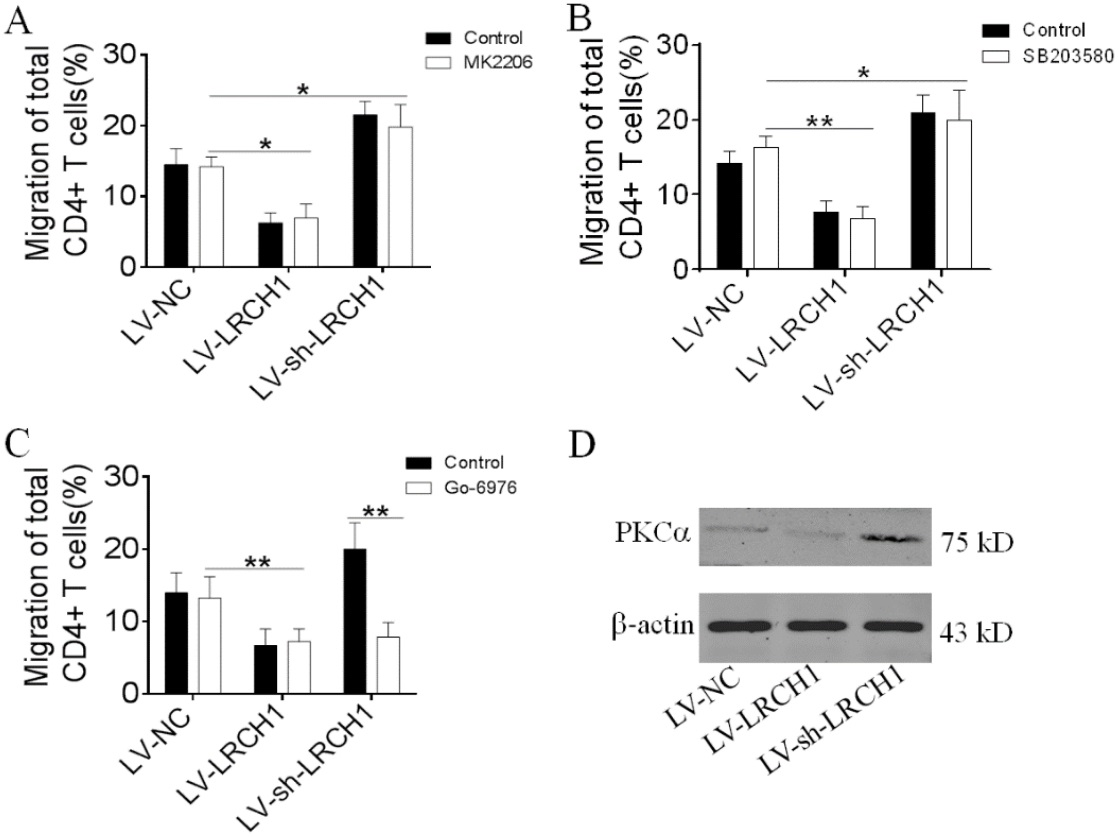
LRCH1 inhibits CD4^+^ T cells migration by PKCα. A-C. Transfected CD4^+^ T cells (1×10^5^, shown in Figure 4) were pretreated with MK2206 (2 μm, Akt inhibitor, A), SB203580 (10 μm, MAPK p38 inhibitor, B) and Go-6976 (1 μm, PKCα inhibitor, C) for 30 mins, and then were used to the chemotaxis assay, as shown in Figure 5. *p < 0.05, **p < 0.01. D. Expression of PKCα in CD4^+^ T cells transfected with LV-LRCH1 or LV-sh-LRCH1 was examined by Western blot. Data are representative of 3 independent experiments.

Several signaling pathways (e.g., MAPK p38, Erk and PKCα) have been reported to be involved in the process of CD4^+^ T cells migration 27, 28. Therefore, we wonder to investigate which mechanism does LRCH1 regulate migration of CD4^+^ T cells. Peripheral CD4^+^ T cells were transfected with LV-LRCH1 or LV-sh-LRCH1, and then were pretreated with MK2206 (2 μm, Akt inhibitor), Go-6976 (1 μm, PKCα inhibitor), and SB203580 (10 μm, MAPK p38 inhibitor) for 30 mins. After 4 h of incubation in Transwell plate, we found that MK2206 and SB203580 didn't affect the migration of CD4^+^ T cells transfected with LV-LRCH1 or LV-sh-LRCH1 (Figure 6A, B). However, treatment with Go-6976 could markedly enhance the migratory capacity of CD4^+^ T cells transfected with LV-sh-LRCH1. There was no significant difference between CD4^+^ T cells transfected with LV-LRCH1 with or without treatment of Go-6976 (Figure 6C). Then we examined expression of PKCα in CD4^+^ T cells transfected with LV-LRCH1 or LV-sh-LRCH1, and we found that PKCα levels were markedly decreased in CD4^+^ T cells transfected with LV-LRCH1, whereas significantly increased in CD4^+^ T cells transfected with LV-sh-LRCH1 compared with controls (Figure 6D). Taken together, these data indicate that LRCH1 may participate in the modulation of CD4^+^ T cells migration through PKCα.

## Discussion

The main characteristics of UC are multiple inflammatory responses associated with mucosal damage, increased epithelial permeability, and massive recruitment of immune cells (e.g., CD4^+^ T cells, macrophages, neutrophils). In this study, we found that LRCH1 expression was highly decreased in colonic mucosa and PBMCs from patients with A-UC, and negatively correlated with disease activity. Up or down regulation of LRCH1 did not affect the differentiation of CD4^+^ T cells, and the related cytokines expression. Moreover, LRCH1 inhibited migratory capacity of CD4^+^ T cells toward CXCL12 by PKCα. Taken together, LRCH1 plays an important role in the pathogenesis of UC, possibly through modulating the migration of CD4^+^ T cells. Therefore, targeting LRCH1 might serve as a novel therapeutic approach in the management of UC.

UC is a chronic and idiopathic inflammatory disease, characterized by relapsing and remitting mucosal inflammation, starting in the rectum and extending to proximal segments of the colon 29. In inflamed colonic mucosa, large amounts of inflammatory immune cells are recruited and flooded into the inflamed mucosal tissues, such as neutrophils, macrophage, natural killer cells, T cells and B cells 6, 30. The disequilibrium between pro- and anti-inflammatory cytokines and increased infiltration of activated immune cells in intestinal mucosa further aggravate mucosal inflammation in UC 31. Genome-wide association studies (GWAS) have identified dozens of loci and genes associated with UC 32, 33. We examined several genes expression in colonic mucosa and peripheral blood cells, we found that expression of one gene, termed as LRCH1, was markedly downregulated in colonic mucosa and peripheral blood cells from UC patients compared with HC.

LRCH1, belonging to the LRCH proteins family, contains LRRs motifs and CH domains, and is widely expressed in heart, spleen, thymus, and intestine 34. LRR motifs display a rapid diversification and have been implicated in protein-ligand and protein-protein interactions 35, 36. CH domains are well established as protein domains mediating interaction with actin filaments 34, 37. Previous studies have demonstrated that LRCH1 is one risk factor of osteoarthritis 19. Our study found that decreased expression of LRCH1 in inflamed colonic mucosa was negatively correlated with Mayo index and UCEIS, which were the representative of clinical and endoscopic activity of UC, respectively. Then, we collected parameters of CRP and ESR, which, to some extent, could reflect the severity of colonic inflammation. Interestingly, expression of LRCH1 in PBMCs from UC patients also was significantly associated with CRP and ESR. These data indicate that LRCH1 might participate in the pathogenesis of UC, however, the mechanisms by which are still obscure.

IFN-γ-producing Th1 cells, IL-4-producing Th2 cells, IL-17A-producing Th17 cells, and Treg cells are reported to be essential for intestinal mucosal homeostasis in the pathogenesis of UC 13, 38. Our published data have demonstrated that Tripartite motif-containing (TRIM) 21 and Rho-associated kinase 2 influenced IBD CD4^+^ T cells to differentiate into Th1, Th17 and Treg cells 14, 15. Therefore, we wondered whether LRCH1 participated in the pathogenesis of UC through modulating the differentiation of CD4^+^ T cells. However, we found that up or downregulation of LRCH1 in UC-CD4^+^ T cells transfected by lentivirus had no effect on the differentiation of CD4^+^ T cells. And there were no significant differences of associated cytokines expression in LV-LRCH1 or LV-sh-LRCH1 transfected CD4^+^ T cells. These data suggest that LRCH1 might be involved in UC development through other mechanisms.

UC is characterized by massive infiltration of immune cells into the mucosa, especially effector T cells. We found that LRCH1 did not participate in the regulation of differentiation, proliferation, and apoptosis of CD4^+^ T cells. Then we wondered whether LRCH1 modulated the migratory capacity of CD4^+^ T cells. We found that upregulation of LRCH1 significantly suppressed the migration of CD4^+^ T cells toward CXCL12, and downregulation of LRCH1 markedly enhanced the migratory capacity of CD4^+^ T cells. CXCL12, a ubiquitous and constitutive chemokine, is reported to mediate T lymphocyte migration to inflamed tissues through its receptor CXCR4, and blockade of CXCR4 mitigates exhaustion of CD4^+^ T cells during sepsis 26, 39, 40. However, in our study, we found that LRCH1 did not regulate CXCR4 expression in CD4^+^ T cells. Previous studies have reported that MAPK p38, Akt and PKC have been involved in the modulation of CD4^+^ T cells migration 20, 27, 28, 41. To investigate how LRCH1 regulate the migration of CD4^+^ T cells, we treated CD4^+^ T cells with inhibitors of Akt, PKCα and MAPK p38. Interestingly, we found that Akt and MAPK p38 did not participate in the process of LRCH1 modulating the migration in CD4^+^ T cells, whereas Go-6976 (a PKCα inhibitor) could markedly enhance the migratory capacity of CD4^+^ T cells with downregulated expression of LRCH1. However, in CD4^+^ T cells with upregulated expression of LRCH1, Go-6976 had no significant effects. Moreover, PKCα levels were markedly decreased in CD4^+^ T cells transfected with LV-LRCH1, whereas significantly increased in CD4^+^ T cells transfected with LV-sh-LRCH1 compared with controls. These data indicate that LRCH1 might suppress expression of PKCα in CD4^+^ T cells, and participate in the process of regulating the migration of CD4^+^ T cells through PKCα.

Taken together, our study demonstrates that LRCH1 expression is highly decreased in UC patients, and negatively correlated with disease activity. Moreover, LRCH1 functions as a novel indispensable regulator in the pathogenesis of UC by suppressing the migration of CD4^+^ T cells through PKCα. Therefore, LRCH1 may play a crucial role in the intestinal inflammation of UC, and LRCH1 may be served as a new therapeutic target for the management of UC.

## Supplementary Material

Supplementary figures and tables.

## Figures and Tables

**Table 1:** The primers using in qRT-PCR analysis.

Gene	Species	DNA sequence (sense 5'-3')	DNA sequence (anti-sense 5'-3')
LRCH1	Human	ACTCTGCACCCACTTCATCAT	GGTACGGGGAAATTCCTTCAAT
T-bet	Human	GTCCAACAATGTGACCCAGAT	ACCTCAACGATATGCAGCCG
GATA3	Human	GCCCCTCATTAAGCCCAAG	TTGTGGTGGTCTGACAGTTCG
RORC	Human	GTGGGGACAAGTCGTCTGG	AGTGCTGGCATCGGTTTCG
Foxp3	Human	GTGGCCCGGATGTGAGAAG	GGAGCCCTTGTCGGATGATG
CXCR4	Human	ACTACACCGAGGAAATGGGCT	CCCACAATGCCAGTTAAGAAGA
GAPDH	Human	CTGGGCTACACTGAGCACC	AAGTGGTCGTTGAGGGCAATG
