# Supplementary material for: LRCH1 suppresses migration of CD4+ T cells and refers to disease activity in ulcerative colitis
Source: Int J Med Sci. 2020 Feb 17;17(5):599–608. doi: 10.7150/ijms.39106 (PMC7085219; doi:10.7150/ijms.39106)
Supplement: Supplementary file 1 — Supplementary figures and tables. [file ijmsv17p0599s1.pdf]

Supplementary Table 1. Clinical characteristics of patients with UC

	Blood samples		Biopsy samples	
	HC	UC	HC	UC
**Number of patients**	26	33	23	30
**Age (y)**	30.35±7.68	37.97±12.42	33.42±10.32	41.32±6.34
**Gender**				
Male	15	14	10	17
Female	11	19	13	13
**Disease duration (month)**		35.42±12.42		39.42±8.53
**Current therapy**				
5-aminosalicylates		17		20
Biologics		0		0
Azathioprine		0		0
methotrexate		0		0
prednisolone acetate		2		4
methylprednisolone		5		6
**Disease extent (UC)**				
E1		7		6
E2		10		9
E3		16		15
**CRP (mg/L)**		38.87±9.08		36.89±19.76
**ESR (mm/h)**		30.64±16.78		25.79±12.79

According to the Montreal classification system. A/R: Active/Remission

Supplementary figure legend

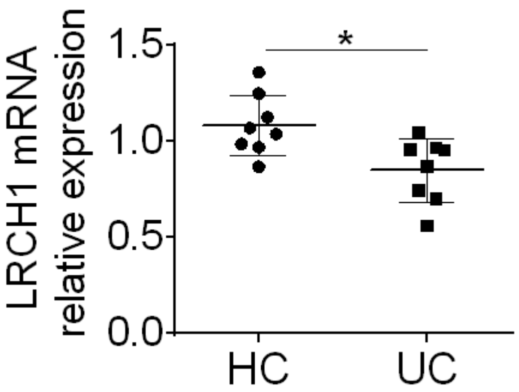

Supplementary Figure 1. $CD4^+$ T cells were isolated from peripheral blood of patients with UC (n = 8), or HC (n = 8), and LRCH1 expression in $CD4^+$ T cells was examined by qRT-PCR. *$p < 0.05$. Data are pooled from 3 independent experiments.

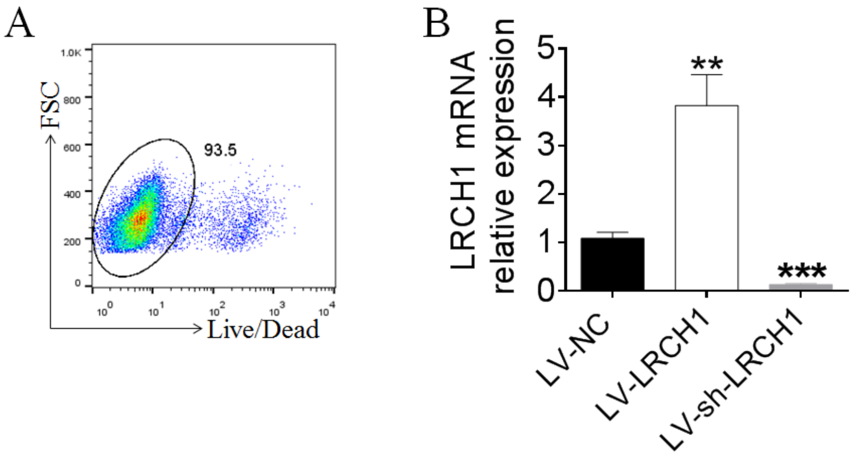

Supplementary Figure 2. A. After 5 h' virus transfection, CD4^{+} T cells were collected, and were used to determine the survival rates by flow cytometry. B. RNA was extracted from transfected cells from HC, and expression of LRCH1 mRNA was determined by qRT-PCR. **$p < 0.01$, ***$p < 0.001$. Data are pooled from 3 independent experiments.

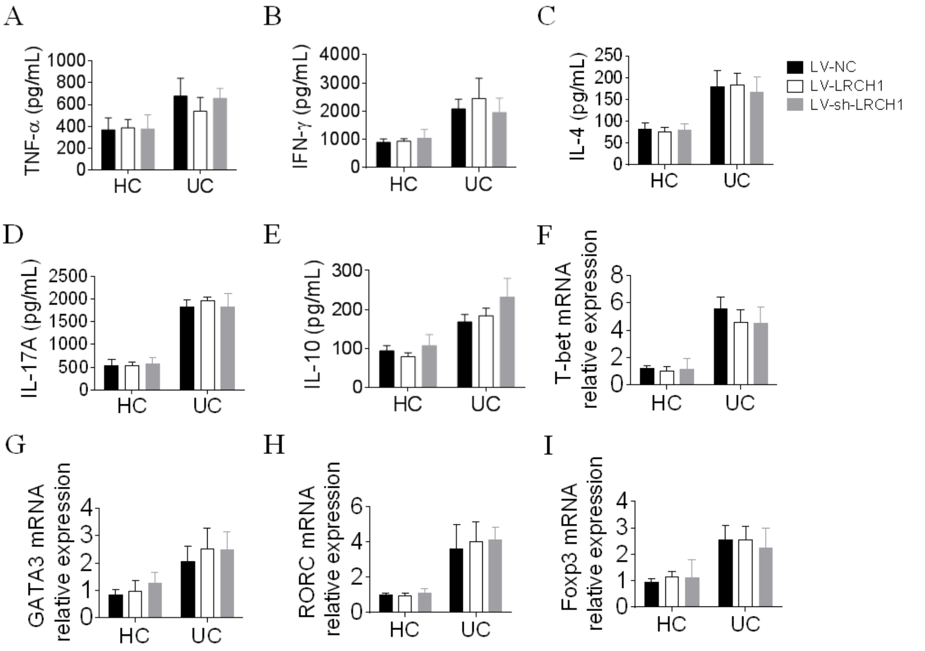

Supplementary Figure 3. LRCH1 does not affect cytokines expression in CD4$^+$ T cells. As shown in Figure 4, cultured cells were also collected on day 5 to determine mRNA expression of T-bet, GATA3, RORC, and Foxp3 by using qRT-PCR (A - D). GAPDH was used as a housekeeping gene. Data are representative of 3 independent experiments. On day 3, supernatants were collected to determine levels of TNF-α, IFN-γ, IL-4, IL-17A, and IL-10 by means of ELISA (E-I). Data are representative of 3 independent experiments.

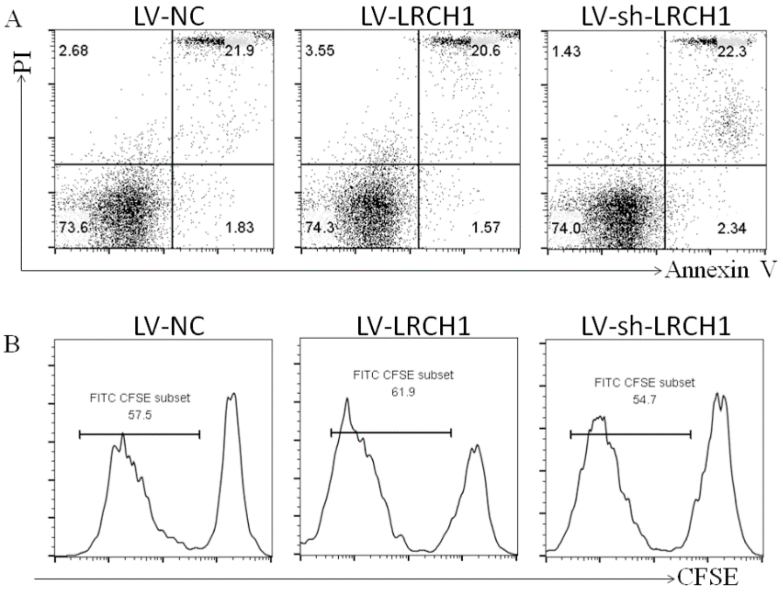

Supplementary Figure 4. A. As shown in Figure 4, cultured CD4$^+$ T cells were collected on day 5 and detected for apoptosis by flow cytometry. B. After the transfection of lentivirus of CD4$^+$ T cells, these cells were then labeled by CFSE (2.5 μM) and cultured under anti-CD3/CD28 stimulation for 5 days. The proliferation of CD4$^+$ T cells was determined by flow cytometry. Numbers on hollow histograms represent the percentage of proliferating T cells.

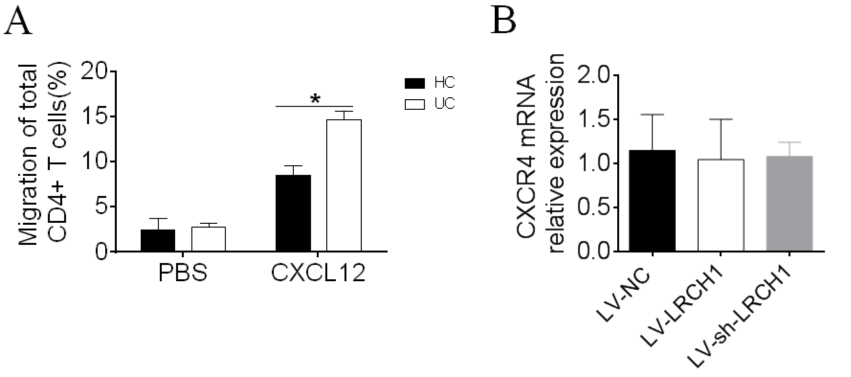

Supplementary Figure 5. A. $CD4^+$ T cells were isolated from peripheral blood of patients with UC and HC, and used to the chemotaxis assay. $CD4^+$ T cells (1×10^5) were suspended in 100 µL medium were placed into the top well of a Transwell chamber (5 µm, Corning), and 600 µL medium containing human CXCL12 (50 ng/ml) was added into the bottom well. After 4 h of incubation, cells in the bottom well were collected and counted. $*p < 0.05$. Data are representative of 3 independent experiments. B. CXCR4 expression was examined in transfected $CD4^+$ T cells (shown in Figure 4) by qRT-PCR. GAPDH was used as a housekeeping gene.
